# The complex social, cultural and psychological drivers of the ‘chemsex’ experiences of men who have sex with men: a systematic review and conceptual thematic synthesis of qualitative studies

**DOI:** 10.3389/fpubh.2025.1422775

**Published:** 2025-02-06

**Authors:** Edward Mundy, Alexander Carter, Tom Nadarzynski, Christopher Whiteley, Richard O. de Visser, Carrie D. Llewellyn

**Affiliations:** ^1^School of Psychology, University of Kent, Canterbury, United Kingdom; ^2^Sussex Partnership NHS Foundation Trust, Worthing, West Sussex, United Kingdom; ^3^Department of Primary Care and Public Health, Brighton & Sussex Medical School, University of Sussex, Falmer, United Kingdom; ^4^School of Social Sciences, University of Westminster, London, United Kingdom; ^5^Central and North West London NHS Foundation Trust, London, United Kingdom

**Keywords:** chemsex, sexualised drug use, polydrug use, substance use, men who have sex with men, qualitative, systematic review

## Abstract

**Introduction:**

Chemsex’ is the sexualised use of drugs among men who have sex with men (MSM). Past systematic reviews have primarily focussed on quantitative research exploring the key characteristics and health consequences of chemsex. However, a large body of qualitative literature exists, drawing on different theoretical frameworks.

**Methods:**

A systematic review and thematic synthesis of the qualitative research on chemsex was conducted with the aim of exploring the chemsex experiences of MSM in the context of substance misuse and addictions, and to reviewing their underlying theoretical frameworks. Six databases were searched, and 43 papers were included in the review.

**Results:**

The thematic synthesis resulted in four key themes: ‘characterising chemsex’, ‘the context around chemsex’, ‘the chemsex experience’, and ‘harms, saying safe, and stopping chemsex’. Only nine papers explicitly drew on theoretical frameworks, which were broadly divided into two categories: those which drew on psychological theories, and those that framed chemsex as a social and cultural phenomenon.

**Discussion:**

The results of the thematic synthesis add further understanding of the key characteristics of chemsex as well as some of the complex social and psychological drivers which may shape why people have chemsex. Finally, the review highlights the clinical implications and inherent complexities in providing clinical services for those reporting chemsex and the need for greater application of theory to advance our understanding of chemsex and continue to develop appropriate forms of therapeutic support.

**Systematic review registration:**

https://osf.io/j6k9r/.

## Introduction

‘Chemsex’ has been defined as the sexualised use of drugs, in particular mephedrone, *γ*-hydroxybutyrate (GHB)/γ-butyrolactone (GBL) and crystallised methamphetamine, most commonly associated with men who have sex with men (MSM) (1, 2). Since the mid-2010s academic interest has increased in response to the emergence of chemsex among MSM (2) and its potential role in increasing rates of sexually transmitted infections (STIs) (3). Previous systematic reviews initially focussed on characterising chemsex as a phenomena and quantifying the health outcomes of people who engaged in chemsex, culminating in several reviews documenting which drugs were used, proximal antecedents to chemsex, motives for chemsex, and the health consequences, particularly in relation to HIV/STIs, other blood born viruses, illicit drug use, and psychological distress (4–6).

In addition to the quantitative research into chemsex, a body of qualitative research has emerged (3, 7–13). This research has explored the experiences of MSM who engage in chemsex, to understand the psychological, social, and cultural, factors that influence chemsex among some MSM. In doing so, this literature has sought to expand the focus beyond a narrow health and risk paradigm (8, 11, 14, 15, 65). Various theoretical and disciplinary approaches have been applied to the study of chemsex, drawing on sociological, cultural, public health, and psychological theory and methods.

There has been no review of the now substantial body of qualitative research into the experiences of MSM who engage in chemsex. Understanding the chemsex experiences of MSM (we use the term MSM to refer to men’s behaviour, and therefore include people who identify as gay, bisexual, heterosexual, or any other way) may contribute to our understanding of how, when, why, and what happens when people engage in this activity. Although previous systematic reviews (4–6, 66) have incorporated qualitative research, they have been limited in their depth of analysis and narrower focus on health consequences associated with chemsex. Furthermore, they have not integrated how different theoretical perspectives have conceptualised chemsex as a phenomenon.

A systematic review and thematic synthesis of the qualitative research on chemsex was conducted. The aims were to explore the chemsex experiences of MSM in the context of substance misuse and addictions, and to review their underlying theoretical frameworks.

## Methods

The study protocol for this systematic review and narrative synthesis of qualitative studies of chemsex experiences of MSM followed PRISMA guidelines, and was registered on Open Science Framework (osf.io/j6k9r).

### Search strategy

Web of Science, ASSIA, Pubmed, PsycINFO, SCOPUS, and EMBASE, were searched using a comprehensive search strategy. Searches of Google Scholar were also undertaken using the terms ‘chemsex’ and ‘qualitative’, alongside citation tracking and hand searching the reference lists of relevant literature. Databases were searched from 2000 onwards, as the early 2000s was when the term ‘chemsex’ is argued to have emerged (2, 16, 17). Databases were searched in March 2021 (by EM), with an updated search in November 2022 (by AC) to identify new papers.

### Inclusion criteria

The review included peer-reviewed studies that explored the experiences of MSM who engage in chemsex. Participants were aged 16+, male, trans or of diverse gender identity, and had engaged in chemsex with other males, trans or people with diverse gender identities. Chemsex was defined as the use of illegal substances, in particular mephedrone, GHB/GBL, and crystallised methamphetamine, for sexual purposes or concurrently with sexual behaviour. However, given the lack of agreed terminology within the literature and geographic and cultural differences in the use of language around sexualised drug use, the review included studies which used a range of terminology, including chemsex, sexualised drug use, party’n’play, slam sex, and ‘chill out’. Furthermore, although chemsex is associated with other substances, such as alcohol, cannabis, amyl nitrate, or sildenafil, studies which reported on these substances were only included if they were used in addition to the focal chemsex substances listed previously. Studies were included if they provided qualitative data from either mixed-methods or qualitative studies and were published in English in peer-reviewed journals.

### Exclusion criteria

Studies were excluded if they did not contain primary data, explored drug use among MSM outside of a sexualised context (i.e., general drug use in MSM); or studied chemsex in non-MSM populations. Studies which adopted a purely quantitative methodology, did not allow meaningful extraction of qualitative data, or were non-peer reviewed (i.e., grey literature) were also excluded.

### Screening procedure

All studies identified from the search strategy were imported into Mendeley with duplicates removed. The database was then shared with each member of the research team. From there, study selection occurred over two phases for each search. During stage one, EM independently screened articles by title and abstract, guided by the eligibility criteria. At the same time, the database was divided equally between TN, CW, and CL who then independently screened articles by title and abstract, guided by the eligibility criteria. Independent evaluations of whether to include papers were shared with the whole team; discrepancies or disagreements were discussed until consensus was reached.

**Figure 1:**
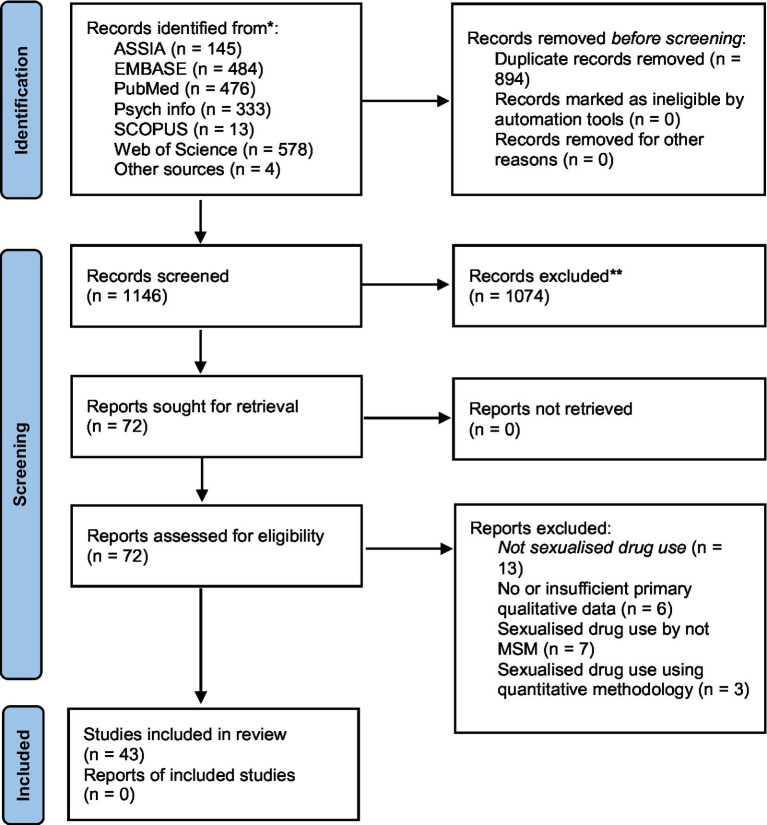
Prisma diagram.

The second stage was independent full-text review of the included studies against the eligibility criteria by EM, TN, CW, and CL. Independent evaluations of whether to include papers were shared with the whole team; discrepancies or disagreements were discussed until consensus was reached. When the updated searches were conducted, the same stepwise procedure was followed with AC carrying out the initial screening. Figure 1 presents a flow diagram.

### Data extraction

Data from eligible studies was extracted and entered into a customised template. Data included key descriptive information (authors, study year, location), study aims and objectives, participants and contextual information (demographic and descriptive information about participants), theoretical framework, methodological information (recruitment, data collection and analysis methods), and results (key findings, main themes and sub-themes, illustrative quotes, and interpretive codes). Data were then imported into NVivo 12 Pro (QSR International Pty Ltd., 2018) for data analyses.

### Data analysis

Data were analysed following the three stages of thematic synthesis described by Thomas and Harden (18). Although these stages are described linearly, this was an iterative process, with stages one and two beginning concurrently, with greater differentiation as the analysis continued and themes were created. Stage one consisted of the systematic line-by-line coding of data (i.e., main themes, sub-themes, illustrative participant quotes) and ascribing meaning to sections of text. The cumulative process of line-by-line coding led to categorisation of findings which were felt to be similar in content into preliminary themes, stage two. Finally, these themes were hierarchically ordered to present the final themes.

### Quality appraisal

Studies were assessed using the Joanna Briggs Institute (JBI) quality appraisal tool for qualitative research (67). The JBI checklist is a widely-used tool that allows researchers to assess the quality of qualitative studies (19). All papers were assessed by AC and their quality was considered when describing and interpreting the results.

## Results

We initially identified 2,040 papers. Following the removal of duplicates and the exclusion of papers that did not meet inclusion criteria on the basis of screening their titles and abstract, 72 papers were retrieved and a full text review was undertaken. Of those, 29 were not included (Figure 1), leaving a final 43 papers for inclusion in the review.

### Study characteristics

Table 1 provides a summary of studies. Most were conducted in the USA (10), the UK (9), Western Europe (11), and Canada (5), alongside studies in Australia (3), South Africa (1), Singapore (1), China (1), Thailand (1) and Malaysia (1). In total, 1,295 participants were included, with sample sizes ranging from 6 to 89. Participants were aged 18–74 and were either engaging in chemsex at the time of the study, or had done so previously. Studies predominantly employed individual interviews and used various analytic approaches: Thematic Analysis (21), Interpretive Phenomenological Analysis (2), Critical Discourse Analysis (2), Content Analysis (4), Grounded Theory (6), Labovian Narrative Analysis (1), Conjectural analysis (1), Ethnography (1), Framework Analysis (1). Four studies contained insufficient detail to allow categorisation of their data analysis (20–22, 23). Four studies used focus groups alongside individual interviews (7, 9, 21, 23).

**Table 1:** Summary of included studies.

Study	Country	Aim	Sample	Data collection period	Qualitative methodology	Theoretical orientation	Key findings
Ahmed et al. (7)	London, UK	To understand the social norms associated with sexualised drug use	Individual interviews with 30 MSM 21–53 years (mean 36) 13 HIV+ 17 HIV-at last test Focus groups with 12 MSM Range 25–53 years (mean 38) 1 HIV+ 11 HIV-at last test	October 2013 and February 2014	Thematic analysis	Positions chemsex as arising, at least in part, out of wider social attitudes around use of drugs and sex (70). Perceptions of popularity and acceptability may motivate individuals to engage in chemsex.	Four key themes were found across individual and focus group data: ‘Ubiquity of Chemsex’, ‘Settings and Spaces’, ‘Permissions and Expectations’, and ‘Drawing a Line’.
Balán et al. (48)	New York, USA	To better understand Latino MSM who engage in intentional condomless sex, including the role of substance misuse as a mediating factor.	Individual interviews with 31 MSM Mean age of 30 9 HIV+ 22 HIV-at last test	April 2005 to March 2006	Thematic analysis	Bareback sex, facilitated for some by substances, may arise out of cultural meanings associated with being a Black or Latino man, in particular linking a sense of greater masculinity to risky behaviours.	Use of methamphetamine associated within a voracious sexual appetite and multiple sexual partners
Bourne et al. (3)	London, UK	To understand the perceived harm associated with chemsex and the harm reduction needs of those who engage in chemsex	Individual interviews with 30 MSM 21–53 years (mean 36) 13 HIV+ 17 HIV-at last test	October 2013 and February 2014	Thematic analysis	Broadly framed within the public health literature raising concerns around the risk to health associated with chemsex, including increased risk of STI/HIV infection and overdose.	Drugs were associated with enhanced sexual experience, but concerns were raised around lack of understanding of how to use drugs safely, participants had experienced overdoses and had concerns around navigating consensual sex when intoxicated, and many reported harm to their physical and mental health. Seeking support for their chemsex use was complicated by concerns around judgement by professionals.
Bourne et al. (36)	London, UK	To understand risk of STI/HIV transmission associated with sexualised drug use	Individual interviews with 30 MSM 21–53 years (mean 36) 13 HIV+ 17 HIV-at last test	October 2013 and February 2014	Thematic analysis	Broadly framed within the public health literature raising concerns around the risk to health associated with chemsex, including increased risk of STI/HIV infection.	Behaviours thought to increase risk of STI/HIV during chemsex varied for participants, with some able to maintain safe sex/drug use practises and others not as a result of their use of drugs. For those who were HIV positive, chemsex did not affect their willingness to engage in condomless anal sex. Chemsex was generally associated with sex that was longer in duration and with more partners.
Chartier et al. (49)	San Francisco, USA	To understand the meaning, personal values, and impact of drug use on HIV-positive men who use methamphetamine	Individual interviews with 22 Gay (18), bisexual (3) and straight (1) man. All HIV positive (years since diagnosis ranged from <1 to 20>)	NS*	Grounded theory	Broadly framed within the public health literature raising concerns around the risk to health associated with chemsex, including increased risk of STI/HIV infection	Methamphetamine was associated with increased sexual appetite, more sex, and more sexual partners
Choi et al. (24)	Hong Kong, China	aimed to describe HIV-negative MSM’s experiences with app usage, the sexual activities arranged accordingly and their experiences in using dating apps to arrange sexual encounters.	31 MSM Mean age 27 (range 18–39 years)	NS*	Thematic analysis	Affordance theory (Gibson, 1979) was used to explain how use of dating apps afforded unique ways of accessing sexual partners	Chemsex was perceived as growing in popularity and associated with condomless anal and group sex. Participants reported feeling in control when it came to chemsex.
Currin et al. (50)	Oklahoma, USA	To explore substance related sex expectancies in the context of more socio-politically conservative areas	Individual interviews with 40 MSM Mean age of 33 years	NS*	Thematic analysis	Broadly framed within the public health literature raising concerns around the risk to health associated with chemsex, including increased risk of STI/HIV infection.	Participants reported that methamphetamine was associated with increase sexual stamina and increased arousal. Marijuana helped enhanced participant’s sexual experiences by enabling them to focus either on the experience and feelings of the sexual act or on their sexual partner. Many described a sensation that marijuana allows a person to quiet worries of the outside world and focus on the sexual experience.
Deimel et al. (51)	Berlin, Cologne, Frankurt/Main, Germany	This study examines reasons for drug use and drug use contexts for MSM, including syndemic factors and experiences of social support	14 MSM Ranged in age from 26 to 60 years	February to April 2015	Content analysis	Chemsex framed as a potential risk to health, either through sexually transmitted infections or through drug use.	The study shows that drug using MSM exhibit additional health-related burden. Their motives for drug consumption are manifold. Besides an improved sexual performance, the coping of various problems play a key role. The consumption of drugs is associated with a risky sexual behaviour
Dennermalm et al. (52)	Berlin, Germany	To improve understanding of drug use, sex on drugs and harm reduction tech-niques among Swedish MSM who travel to Berlin in order to improve health among MSM using drugs	15 Swedish MSM men Ranged in age from 23 to 44 years	January 2016 to June 2017	Thematic analysis	Broadly framed within the public health literature raising concerns around the risk to health associated with chemsex, including increased risk of STI/HIV infection.	There is a need for increased adoption of harm reduction techniques in this population focusing on mitigating harm and prevention of risk of problematic use or starting injection drugs. Existing traditional services require adaptations to become more accessible and acceptable to sub-groups of drug users, including low-threshold services providing non-judgemental, evidence-based information. This will require funding of alternative providers such as STI/HIV clinics, among others, and health care providers to increase adoption of prevention strategies, specifically pre-exposure prophylaxis for HIV
Dew(23)	Atlanta, USA	To investigate context factors that influence methamphetamine use among non-addicted MSM	Focus group: 20 non-addicted methamphetamine-using MSM Individual interviews 10 non-addicted methamphetamine-using MSM Mean age 40.2 years (range: 24–56)	October 2008 and February 2009	Broadly TA/inductive	Broadly framed within the public health literature raising concerns around the risk to health associated with methamphetamine use	Participants reported that a key motivator for using methamphetamine is its ability to enhance sexual pleasure. This had additional physiological benefits such as improving arousal, stamina, and ability to maintain an erection. There were also psychological benefits including reducing negative feelings of guilt and shame associated with sex, increased disinhibition, and improved self-confidence.
Drysdale et al. (8)	Melbourne, Perth, Sydney, Australia	To explore wider context associated with ‘chemsex’ to highlight the variability and contingencies of how chemsex is practised	Individual interviews with 88 gay and bisexual men Median age of 45 (range 21–74 years).	September 2017 and July 2018	Grounded theory	Critical of the wider literature on chemsex which is concerned with ‘risky’ sexual behaviour that may result from drug use. In doing so, chemsex has been framed within a narrow and unidirectional focus on drugs as a mediator of risky sexual behaviour, ignoring the variability in practise, settings, and outcomes.	Findings from the project revealed that men used crystal in a variety of settings and relations, which mediated their sexual practises and patterns of use. In looking at the wider context in which practises were associated with the combination of sex and drugs, we identified experiences that the contemporary discourse of chemsex—in its rhetorical proposition of at-risk behaviours and circumstances—may leave out of consideration.
Flores-Aranda et al. (9)	Montreal, Canada	To explore perceptions and experience of pleasure in relation to chemsex	Focus group with 10 MSM currently using methamphetamine Focus group with 8 MSM who are no longer using methamphetamine 15 gay, 2 bisexual. Average age of 35 years (24–55).	NS*	Thematic analysis	Framed within the public health literature citing increase in substance misuse within MSM and increased risk of HIV/STIs. Critical of approach which has not explored the role of pleasure as a key motivating factor.	Use of methamphetamine closely associated with experiences of heightened pleasure, including in relation to sex. Four key themes of the pleasure were identified: the pleasure associated with sexual practises and their effects, the pleasure associated with one’s relationship to oneself and to others; the pleasure associated with injection; and the decrease in pleasure associated with the consequences of substance use and the necessity of service use
Gaissad and Velter (32)	Paris, France	To describe the sexual practises involved in chemsex with the intention of highlighting how drugs are used for sexual purposes.	33 MSM 22–61 years old	NS*	Ethnographic	Critical of research which has largely framed chemsex in the context of health risk, in the process misses a core motivating factor which is the use of drugs for sexual purposes.	Four key themes emerged from their findings: a division within the paris gay scene between those who used drugs for sexual purposes and those who used them for clubbing; chemsex as a shared experienced of people coming together; chemsex enhanced physical performance when having sex and amplified pleasure; chemsex helped reduce inhibitions and allowed people to “let go”;
Graf et al. (10)	Berlin, Cologne, Frankurt/Main, Germany	Describe the perceived prevalence of chemsex in Germany and understand the motivations and consequences of sexualised drug use	89 MSM 22–64 years old	NS*	Content analysis	Chemsex framed as a potential risk to health, either through sexually transmitted infections or through drug use.	Chemsex was seen as becoming increasingly prevalent on the gay scene in Berlin, Cologne, and Frankfurt/Main. However, recognition that negative health outcomes varied with some able to engage in chemsex in safe and manageable way. Primary motivation for chemsex was to intensify sexual feelings and performance, reduce inhibitions, and improve feelings of self-worth. Problematic use was associated with overdosing, reduced wellbeing, difficulties practising safe sex, and acquiring sexually transmitted infections.
Green and Halkitis (53)	New York City, USA	To understand social factors associated with crystal methamphetamine	49 gay and bisexual men who use crystal methamphetamine	January–February 2001	Grounded Theory	Critical of how psychological, health, and epidemiology have focused on individual factors in explaining use of crystal methamphetamine by gay and bisexual men. In doing so, this has ignored important social factors; in particular how those who engage in sexualised drug are represent a form of “sexual sociality” a coming together of people within a specific context and purpose which is infused with wider social norms which interact and shape why and how drugs are used.	Crystal Methamphetamine was associated with reducing in sexual inhibitions, increased libido, improve sexual performance, improved self-esteem, increase pain threshold, used to navigate sexual sociality, and increased sexual pleasure
Guadamuz and Boonmongkon (54)	Thailand	To explore the meaning, risks, social contexts, use of internet and mobile technology of young men who have sex with who use ice (crystal methamphetamine) for sexual purposes.	40 MSM who use crystal methamphetamine for sexual purposes Age 18–29	NS*	Grounded Theory	Chemsex, in particular ice parties (use of crystal methamphetamine with others for sexual purposes) was conceptualised as social phenomena in which sexual and gender minorities could meet. Ice parties were a form of ‘secret sociality’ which existed physically in private settings but enabled through internet and mobile technology.	The use of methamphetamine for sexual purposes was infused with culturally specific meanings of exclusivity, ideas of physical beauty, and social status. Methamphetamine was associated with sexual fluidity of roles and practise, heightened pleasure, multiple partners, and largely took place in private settings. There were important distinctions between those who attended parties and hosted, the former often being older in age and supplied drugs. Ice parties could also be associated with social hieratchy, relational power, and sexual violence,
Hakim (11)	London, UK	To explore the emergence of chemsex as a reponse to specific historical material conditions.	15 MSM aged between 24–51	May and June of 2016	Conjectural analysis	Draws on conjectural analysis to frame chemsex as a phenomena which has arisen out of an interaction of historically contingent social forces.	Chemsex is as an attempted by MSM to regain a sense of collectivity and social spaces in the context of increased atomisation of social bonds as a result of the emergence and dominance of neo-liberalism.
Halkitis et al. (20)	New York City, USA	Explore reasons for methamphetamine use among gay and bisexual men	48 MSM currently using methamphetamine Aged 19–49 years old HIV + 27 (56.3%) HIV− 20 (41.7%) HIV Unknown 1 (2.1%)	NS*	Unclear	Framed within the wider literature exploring prevalence of drug use among MSM and concerns about negative health consequences.	Majority of participants reported that they used methamphetamine for sexual purposes. Methamphetamine enabled longer sexual encounters, enhanced pleasure, reduced inhibitions, and increase number of partners.
Hawkins et al. (55)	Vancouver, Canada	Understand how drugs are used for social inclusion among gay, bisexual, and other MSM	20 gay, bisexual, and MSM median age was 35 years (range 21–66 years) 6 (30%) reported living with HIV	August and October 2012	Thematic analysis	Framed within the wider literature exploring prevalence of drug use among MSM and concerns about negative health consequences.	Participants reported different drugs for different social situations. For those who wanted to use drugs for sexual purposes, crystal methamphetamine and cocaine were reported as primary drug of choice.
Herrijgers et al. (56)	Antwerp, Belgium	to understand current risk reduction practises, and the information and care needs of gay, bisexual, and other men who have sex with men (GBMSM) who engage in chemsex	20 GBMSM Mean age of 43 (range, 26 and 69 years old)	January and March 2020	Thematic analysis	Absence of explicit theoretical framework, but study is framed around the public health literature which expresses concern around health risk associated with chemsex and therefore need to understand harm minimisation and health needs of this population.	Participants reported a range of harm reduction strategies and practises. Paricipants also spoke of a range of needs when accessing support as well as barriers to accessing help.
Hibbert et al. (57)	Northern and Central England, UK	to understand MSM and service provider (SP) perspectives of the current standard of service provision for MSM engaging in sexualised drug use	13 MSM Median age of 34 years (range 23–66). 7 service providers	January–December 2019	Thematic analysis	Absence of explicit theoretical framework, but study is framed around the public health literature highlighting increased risk of health risks through sexualised drug use	Participants reported that a primary motivation for sexualised drug use was the enhancement of sexual experiences. Whilst participants were generally satisfied with the support they received from sexual health services, four barriers to access were identified
Jaspal(26)	London and East Midlands, England, UK	To understand the motivations behind chemsex use and the function that the practise plays in constructing a positive sense of self and the managing of psychological distress	16 GBM Aged between 22–47 years old10 participants HIV negative 6 as HIV positive	NS*	Thematic analysis	Identity Process Theory was used to how chemsex use informs the (re)construction of identity.	Three key themes were found around how chemsex shapes identity: (1) identity threat and the allure of chemsex, (2) deflection to cope with identity threat, and (3) chemsex as a gateway to deflection
Joyce et al. (58)	Dublin, Ireland	To explore problematic GHB use as experienced by people who had presented for treatment	7 (5 gay men, 2 females) Men aged 31–39 years	NS*	IPA	Framed within the wider literature exploring prevalence of drug use among MSM and concerns about negative health consequences.	GHB use was used for sexual purposes with individual partners and in group sex parties. GHB associated with enhanced sexual pleasure and reduced inhibitions. Others reported it use for sexual purposes was only one aspect of their GHB use and some reported libido reduced over time. Participants also reported sexual violence and acquiring STIs.
Klitzman (59)	New York City, USA	To explore MDMA use, context, popularity, and perceived side effects among gay men	12 gay men	1999 and 2001	Grounded Theory	Framed within the wider literature exploring prevalence of drug use among MSM and concerns about negative health consequences.	MDMA was associated with increased sexual functioning, disinhibition, and increased desire.
Kubicek et al. (60)	Los Angeles, USA	To understand the positive and negative attitudes toward crystal methamphetamine, cocaine, and ectasy among young MSM	24 gay (74%) and bisexual (16%) men. 12 had used drugs within last 30 days, 12 reported no life time use of any drugs	June–July 2006	Grounded Theory	Framed within the literature exploring ‘club drugs’ in gay community and concerns around potential negative health consequences.	Crystal methamphetamine associated with increase pleasure during sex, however this was not ubiquitous across sample, with some reporting it to interfere with sexual functioning/performance. Ectasy associated with enhacing sensually, both sexually and non-sexually. Drugs also used to help alleviate anxieties in having sex with strangers. Drugs also Cocaine was not mentioned in reference to sex.
Lim et al. (61)	Kuala Lumpur, Malaysia	To understand the motivations for, and management of, methamphetamine use among MSM in Malaysia	20 MSM aged between 21–43 (median age of 34 years) who use amphetamines, including methamphetamine, for sexual purposes	April to December 2014	Thematic analysis	Framed within wider public health literature raising concern around negative health problems risk of drug use in sexual context, including risk to physical and mental health	Primary motivation for drug use was to enhance sexual experience and performance, including increased libido, pleasure, and stamina, and reduced inhibitions. Participants reported both difficulties associated with managing their drug use as well as being able to use the drug safely.
Milhet et al. (33)	Paris, France	To explore the ‘dynamics of pleasure’ in individual experiences of chemsex.	33 MSM aged between 21–61. 17 were HIV positive 21 active chemsex users	NS*	Content analysis	Critical of the ‘pathology paradigm’ which has come to frame chemsex literature. Instead sees those who engage in chemsex as social actors who use drugs for a range of reasons and without necessarily being problematic in outcome.	Pleasure is a complex and multifaceted concept within chemsex. This relates to “incommunicable bodily experiences,” enhanced intimacy and feelings of love, valuing the social aspect of the experience, and a loss of inhibition, psychologically, bodily, and spiritually. Participants also reported a dark side to their chemsex experience, including, increase isolation, addiction, sexual assauly, and sexual frustration.
Nimbi et al. (34)	Italy	To investigate contexts, patterns of substance use, first chemsex experience, and harm reduction in a group of MSM practicing chemsex in Italy	30 MSM with average age of 39.7 years (min 26–62).	February and July 2019	Content analysis	Framed within the public health literature raising concerns around risk to mental and physical health, in particular risk of addiction and STIs	Chemsex was largely defined as the use drugs for sexual purposes; participants reported using multiple substances; participants were introduced to chemsex in a variety of ways; many of the participants were positive toward way of promoting harm reduction initiatives, but not universally.
Nimbi et al. (35)	Italy	To have a better understand-ing of the sexual experience of MSM practicing chemsex under a psycho-sexological biopsychosocial perspective	31 MSM Average age 38.64 (range 26–62)	February and July 2019	Thematic analysis	Framed within the public health literature raising concerns around risk to mental and physical health, in particular risk of addiction and STIs	The promotion of safer sexual behaviour should contemplate an in-depth discussion and recognition of both pleasurable and distressing aspects of chemsex sexual experience, its motivations over time and perceived differences with sober sex
Palamar and Halkitis (62)	New York City, USA	To understand the reasons for GHB use among gay men	15 gay men aged between 24–50.	February 2001 through October 2002	Thematic analysis	Broadly framed within the public health literature identifying increased use of ‘club drugs’ within MSM community and potential negative health risks.	All participants reported that GHB was associated with increased libido and pleasure, decreased inhibitions, and sex with more partners
Parent et al. (31)	Vancouver, Canada	To understand how cannabis is used within the sexual lives of young sexual minority men	41 Median age of 34 (range 18–30).	January and December 2018	Thematic analysis	Framed within the literature raising concerns around negative health risks associated with chemsex whilst acknowledging need to understand chemsex within a broader psycho-social framework	Cannabis use helped to enhance sexual encounters and reduce anxiety and foster intimacy.
Parry et al. (21)	Durban, Cape Town, Pretoria, South Africa	To explore how drug using MSM explain the relationship between drug use and sexual behaviour	Total = 78 N 46 1:1 interviews N 32 focus groups 1:1 interview mean age of 28.9 years (range 18–50), NS for FG but range of 19–55	NS*	Broadly thematic	Framed within the public health literature exploring the potential negative health implications of use of drugs and sex, in particular increased risk of HIV and other sexually transmitted infections.	Drugs were found to facilitate and enhance sexual encounters and increase arousal and could be associated with increased risk sexual behaviour.
Parsons et al. (63)	New York City, USA	To understand the contexts in which young gay and bisexual men were introduced to methamphetamine use	54 gay and bisexual men mean age of 24.9 years (18–29)	December, 2004 and August, 2006	Thematic analysis	Framed within the public health literature raising concerns about negative health risks associated with drug use in MSM, as well as a need to explore the psycho-social motivators of drug use including, loneliness, negative perceptions of attractiveness, aging, and lowering sexual inhibitions.	Methamphetamine was associated with increased sexual aggression (appetite?) as well as increased disinhibition.
Pienaar et al.(22)	Australia	To offer a nuanced analysis of the diverse factors shaping LGBTQ people’s drug consumption, and the varied drug effects that they pursue to transform their experience of gender and/or sexuality	42 MSM Age range: 18–75 years Mean = 38.02 (SD-13.44)	NS*	Inductive approach	Post modernism/structuralist theory of Foucault ‘technologies of self’	Drug use can transform gendered experience and enable the expression of non-normative gender identities, in the process challenging gender binarism
Pollard et al. (25)	London and Brighton, UK	To explore MSM accounts of the role of marginalisation and the psycho-social context of chemsex	15 gay men mean age 33 [range 20–44]	NS*	Framework analysis	Minority stress theory is used to conceptualise chemsex as emerging from and maintained by the syndemics (definition?) of stigma, marginalisation, minority stress, and maladaptive coping.	Six key themes were found within the data relating to (1) the cultural environment of chemsex; (2) intimacy and loneliness; (3) vicious cycles of drug use and sex that participants struggled to understand; (4) social networks and romatic relationships; 5) avoiding, reducing, or stopping drug-use and/or chemsex, and 6) HIV (and STI) risks)
Race et al. (29)	Australia		13 gay men Age ranged from 27 to 66 years	NS*	Thematic analysis	Sexual script theory	Slamming is not merely a technical preference but operates as a constitutive element in a specific kind of sex that practitioners routinely differentiate from, and are inclined to choose over, other kinds of (chem)sex. For participants, knowing that a pro-spective sexual partner was into slamming seemed to be as important a consider-ation as their appearance, location, sexual role and erotic interests
Santoro et al. (15)	Madrid, Spain	To explore and identify different types of chemsex sessions.	11 gay and MSM	September 2018 and February 2019	Thematic analysis and sociological discourse analysis	Drawing on the work of Sociologist Bruno Latour, it is argued that chemsex as a phenomena has been ‘black boxed’ giving a false impression of accepted conceptual clarity. Instead, chemsex needs to be subjected to a more detailed conceptual scrutiny by empirically exploring variation in practise and the hypothesised factors, in particular social norms and trends, which may give rise to chemsex.	four different types of chemsex sessions—“anonymous sessions,” “chill-sex,” “semiclosed parties among networks of friends” and “chemsex in saunas or other sex on premise venues (SPVs)”
Smith and tasker (12)	London, UK	To explore gay men’s narrative of chemsex and recovery following a therapeutic intervention for chemsex	6 Gay men aged between 30–60 years	2015	Labovian Narrative Analysis	Framed within the public health literature raising concerns around potential negative health consequence of chemsex, in particular STIs and HIV. However, the paper seeks to explore contextual psycho-social factors which may lead people to engage in chemsex.	Three central themes within the narratives of participants: (1) acceptance and belonging; (2) life on the verge of collapse; and (3) the uncertainty of a future without chemsex.
Souleymanov et al. (28)	Toronto, Canada	To critically examined the risk and pleasure discourses of gay and bisexual men who PNP	44 MSM Average age of 37 (range 20–69)	NS*	Critical Discourse Analysis	Paper draws on post-structuralist theory, in particular the work of Foucault and Deluz, to explore the risk and pleasure dscourses of gay and bisexual men who engage in party and paly (chemsex). In particular exploring how biomedical discourses may influence or structure perceptions of risk and pleasure.	The findings from this study demonstrated the capacity of biomedical discourses to affect respondents’ HIV risk perceptions and practises. While discourses on risk and pleasure were interwoven within complex PNP assemblages, the notion of pleasure was mobilised as a discursive tactic of self-control, and the division between normative and non-normative pleasures highlighted the consequence of biopolitical forces governing the production of discourses on sex and drugs.
Souleymanov et al. (27)	Toronto, Canada	To problematizes how gay and bisexual men who seek PNP online are able to keep themselves informed about how to choose and conduct their lifestyle in accordance with various discourses that circulate in PNP subcultures.	44 MSM Average age of 37 (range 20–69)	NS*	Critical Discourse Analysis	Draws on post-structural theory, symbolic interactionism, and Bourdie’s social theory to position chemsex within a social, historico-cultural, and linguistic theoretical framework.	The study findings presented evidence for subcultural discourses of gay and bisexual men who seek PNP online, and revealed how these discourses were implicated in recasting the practises of biopolitics, as well as enacting risk subjectivities of these men.
Tan et al. (64)	Singapore	to explore the perceptions of, and reasons for engaging in chemsex among GBMSM in Singapore, and suggest ways to address the harms associated with chemsex in the present setting.	30 Gay, Bisexual, and MSM Age range of 18–39	NS*	Thematic analysis	Framed within the public health literature raising concerns around risk to health associated with chemsex, in particular, increase risk of HIV and other STIs.	Chemsex was seen a common within the GBMSM community in Singapore but were more likely to be used in private spaces. Participants reported three reasons for engaging in chemsex: enchance sexual experience, interpersonal factors, and broader social stressors. The main barriers to addressing risk associated with chemsex relative to the punitive nature of the law around drugs which stopped people seek help.
Van Hout et al. (30)	Dublin, Ireland	to explore sexualised drug use pathways among gay, bisexual and other men who have sex with men experiencing physical and emotional health problems as consequence of their engagement with the sexualised drug use who are seeking help.	10 Gay, Bisexual, and MSM Age range of 35–59	2017	Interpretative Phenomenological Analysis	Framed within the public health literature raising concerns around the negative health risk associated with chemsex, in particular increased risk of HIV, but the need to understand wider social and context factors which may lead someone to engage in sexualised drug use.	Four main themes emerged from the analysis: social and cyber arrangements within the Dublin Chemsex scene; poly drug use and experiences of drug dependence; drug and sexual harm reduction within the Chemsex circle of novices and experts; and sexualised drug use, escapism and compulsive participation. Two additional higher levels of abstraction could be identified above theme level: first, the mutually reinforcing aspects of drug use and sexual pleasure; and second, the interplay between excesses of drugs and sex and dependence
Weatherburn et al. (13)	London, UK	To understand the motivation and values associated with chemsex among gay men	30 gay men Mean age of 36 years (range 21–53) 13 living with a diagnosis of HIV	August 2013 and February 2014	Thematic analysis	Broadly framed within the public health literature raising concerns around potential negative health consequences of chemsex, in particular increased risk of HIV. Also acknowledges need to understand motivating factors in order to develop successful health promotion campaigns	There were two main findings. The first motivation for combining drugs with sex was that drugs provide the means by which men can have the sex they desire by increasing libido, confidence, disinhibition and stamina. The second major motivation for chemsex was that drugs enhance the qualities of the sex that men value. Drugs made other men seem more attractive, increased physical sensations, intensified perceptions of intimacy and facilitated a sense of sexual adventure

*Not stated.

### Risk of bias assessment

All studies were of a high quality. The majority fell down on reporting their theoretical perspective and failing to address the effect that the researcher may have on the participant responses and vice versa. All studies scored highly (ranging from 7 to 9 out of 10) for their methodology and reporting of results, as well as accurate representation of participant voices (Table 2).

**Table 2:** Summary of JBI checklist quality appraisal.

Study	Is there congruity between the stated philosophical perspective and the research methodology?	Is there congruity between the research methodology and the research question or objectives?	Is there congruity between the research methodology and the methods used to collect data?	Is there congruity between the research methodology and the representation and analysis of data?	Is there congruity between the research methodology and the interpretation of results?	Is there a statement locating the researcher culturally or theoretically?	Is the influence of the researcher on the research, and vice-versa, addressed?	Are participants, and their voices, adequately represented?	Is the research ethical according to current criteria or, for recent studies, and is there evidence of ethical approval by an appropriate body?	Do the conclusions drawn in the research report flow from the analysis, or interpretation, of the data?	Overall appraisal	Overall score (out of 10)
Ahmed et al. (7)	Unclear	Yes	Yes	Yes	Yes	No	No	Yes	Yes	Yes	Include	7
Balán et al. (48)	Yes	Yes	Yes	Yes	Yes	No	No	Yes	Yes	Yes	Include	8
Bourne et al. (3)	Unclear	Yes	Yes	Yes	Yes	No	Unclear	Yes	Yes	Yes	Include	7
Bourne et al. (36)	Unclear	Yes	Yes	Yes	Yes	No	Unclear	Yes	Yes	Yes	Include	7
Chartier et al. (49)	Unclear	Yes	Yes	Yes	Yes	No	No	Yes	Yes	Yes	Include	7
Choi et al. (24)	No	Yes	Yes	Yes	Yes	No	No	Yes	Yes	Yes	Include	7
Currin et al. (50)	Unclear	Yes	Yes	Yes	Yes	No	Yes	Yes	Yes	Yes	Include	8
Deimel et al. (51)	No	Yes	Yes	Yes	Yes	No	No	Yes	Yes	Yes	Include	7
Dew (23)	Unclear	Yes	Yes	Yes	Yes	No	Unclear	Yes	Yes	Yes	Include	7
Dennermalm et al. (52)	No	Yes	Yes	Yes	Yes	No	Yes	Yes	Yes	Yes	Include	8
Drysdale et al. (8)	Yes	Yes	Yes	Yes	Yes	No	Unclear	Yes	Yes	Yes	Include	8
Flores-Aranda et al. (9)	Unclear	Yes	Yes	Yes	Yes	No	No	Yes	Yes	Yes	Include	7
Gaissad and Velter (32)	No	Yes	Yes	Yes	Yes	No	No	Yes	Yes	Yes	Include	7
Graf et al. (10)	No	Yes	Yes	Yes	Yes	No	No	Yes	Yes	Yes	Include	7
Green and Halkitis (53)	Yes	Yes	Yes	Yes	Yes	No	Unclear	Yes	Yes	Yes	Include	8
Guadamuz and Boonmongkon (54)	No	Yes	Yes	Yes	Yes	No	Yes	Yes	Yes	Yes	Include	8
Hakim (11)	Yes	Yes	Yes	Yes	Yes	Yes	Yes	Yes	Yes	Yes	Include	10
Halkitis et al. (20)	No	Yes	Yes	Yes	Yes	No	Yes	Yes	Yes	Yes	Include	8
Hawkins et al. (55)	No	Yes	Yes	Yes	Yes	No	No	Yes	Yes	Yes	Include	7
Herrijgers et al. (56)	Yes	Yes	Yes	Yes	Yes	No	No	Yes	Yes	Yes	Include	7
Hibbert et al. (57)	Yes	Yes	Yes	Yes	Yes	No	No	Yes	Yes	Yes	Include	8
Jaspal (26)	No	Yes	Yes	Yes	Yes	No	No	Yes	Yes	Yes	Include	7
Joyce et al. (58)	No	Yes	Yes	Yes	Yes	Yes	Yes	Yes	Yes	Yes	Include	9
Klitzman (59)	No	Yes	Yes	Yes	Yes	No	No	Yes	Yes	Yes	Include	7
Kubicek et al. (60)	No	Yes	Yes	Yes	Yes	No	No	Yes	Yes	Yes	Include	7
Lim et al. (61)	No	Yes	Yes	Yes	Yes	No	No	Yes	Yes	Yes	Include	7
Milhet et al. (33)	No	Yes	Yes	Yes	Yes	No	No	Yes	Yes	Yes	Include	7
Nimbi et al. (34)	No	Yes	Yes	Yes	Yes	No	Yes	Yes	Yes	Yes	Include	8
Nimbi et al. (35)	Yes	Yes	Yes	Yes	Yes	No	Yes	Yes	Yes	Yes	Include	9
Palamar and Halkitis (62)	No	Yes	Yes	Yes	Yes	No	Yes	Yes	Yes	Yes	Include	8
Parent et al. (31)	No	Yes	Yes	Yes	Yes	No	No	Yes	Yes	Yes	Include	7
Parry et al. (21)	No	Yes	Yes	Yes	Yes	No	No	Yes	Yes	Yes	Include	7
Parsons et al. (63)	No	Yes	Yes	Yes	Yes	No	No	Yes	Yes	Yes	Include	7
Pienaar et al. (22)	Yes	Yes	Yes	Yes	Yes	Yes	No	Yes	Yes	Yes	Include	9
Pollard et al. (25)	Yes	Yes	Yes	Yes	Yes	No	No	Yes	Yes	Yes	Include	8
Race et al. (29)	No	Yes	Yes	Yes	Yes	No	No	Yes	Yes	Yes	Include	7
Santoro et al, (15)	No	Yes	Yes	Yes	Yes	No	Yes	Yes	Yes	Yes	Include	8
Smith and Tasker (12)	Yes	Yes	Yes	Yes	Yes	No	Yes	Yes	Yes	Yes	Include	9
Souleymanov et al. (28)	No	Yes	Yes	Yes	Yes	No	No	Yes	Yes	Yes	Include	7
Souleymanov et al. (27)	Yes	Yes	Yes	Yes	Yes	No	No	Yes	Yes	Yes	Include	8
Tan et al. (64)	No	Yes	Yes	Yes	Yes	No	No	Yes	Yes	Yes	Include	7
Van Hout et al. (30)	No	Yes	Yes	Yes	Yes	No	No	Yes	Yes	Yes	Include	7
Weatherburn et al., (13)	No	Yes	Yes	Yes	Yes	No	No	Yes	Yes	Yes	Include	7

### Theoretical frameworks

Only nine studies framed their understanding of and approach to chemsex within an explicit theoretical framework (Table 3). These papers were broadly divided into two categories: those which drew on psychological theories, and those that framed chemsex as a social and cultural phenomenon.

**Table 3:** Theoretical frameworks of studies.

Categories	Studies	Theories	Explanation
Psychological	Ahmed et al. (7)	Social Norms Theory (Sunstein Berkowitz Ajzen)	Social norms—the attitudes and beliefs held by individuals and groups-may play an important role in explaining why people engage in chemsex. Social norms held by communities may influence the decision to behave in certain ways, either through peer pressure or because it may be widely seen as a socially acceptable behaviour.
	Choi et al. (24)	Affordance Theory	Choi (24) uses affordance theory to suggest that geo-spatial social networking apps offer an environment of mobility, proximity, and immediacy that allows for the quick and easy access of sexual encounters, including chemsex.
	Jaspal (26)	Identity Process Theory	Chemsex is a strategy used to deflect threats to identity from the outside world, as well as enhance it through the construction of a more positive identity, the enhancement of self-esteem and self-efficacy.
	Pollard et al. (25)	Minority Stress Theory	Minority stress is the social stresses experienced by a minority group that arise from the experience of marginalisation and stigma. Chemsex may be seen as a means of coping with the stress in that it allows for the psychological alleviation of distress as well as the promotion of spaces where this stress is not experienced.
Cultural studies and sociological	Hakim (11)	Conjectural analysis	Chemsex within London is seen as an attempt to reassert a collective or shared experienced among MSM, which has been undermined as neo-liberalism (free-market capitalism) has become the dominant economic paradigm. Neo-liberalism has resulted in the erosion of gay spaces that fostered a sense of collective identity, facilitated the increase in transient populations, and fostered a wider culture which has promoted an individualisation of gay identity.
	Pienaar et al. (22)	Foucault’s ‘technologies of the self)	Drug use enables the transformation of experience (‘technologies of the self’), particularly in relation to sex, sexuality, and gender.
	Race et al. (29)	Sexual script theory	Sexual scripts provide ‘normative guidelines’ for sexual practises, which are broadly formulaic in nature and act as the basis in which sexual improvisation is enacted. The phenomena of slam-sex results in the development of
	Santoro et al. (15)	Latour’s theory of ‘blac kboxing’	The concept of ‘black boxing’ is used to suggest that the wider literature on chemsex has taken the concept for granted, suggesting that its conceptual parameters and definition has been established. This serves the function of limiting the possibility of scrutiny and exploration of chemsex as a phenomena, ignoring the role of social and cultural practises, change over time, and social and norms and trends.
	Souleymanov et al. (27, 28)	Foucauldian social theory and Deleuzo–Guattarian ‘assemblage theory’	Drawing on the works of Foucault, it is argued that wider social discourses shape the meaning that is attached to behaviours. In the context of sex and drugs, discourses primarily attribute pathology, compulsion, and risk to the use of drugs, particular in relation to sexual practises as the risk of HIV/STIs and health risks associated with drugs. Yet central to drug use and including for sexual purposes, is pleasure, which is typically under acknowledged Assemblage theory is used to highlight the multiple factors which may converge (or assemble) to enable use of sex and drugs, including the role of pleasure and risk.

Papers which explicitly drew on psychological theory used Affordance Theory (24), Syndemic and Minority Stress theories (25), Social Norm Theory (7), and Identity Process Theory (26). In drawing on Affordance Theory (68), Choi et al. (24) view chemsex as emerging through the proliferation and use of digital technology, in particular geo-spatial dating apps, among MSM. Technology allows for the creation of a digital environment which offers users three unique ‘affordances’: (1) mobility in that smart phones can be used at anytime and anywhere by users; (2) proximity both physically and digitally to others who are using the app; and (3) immediacy in terms of ease in arranging sexual encounters. These affordances are given greater salience in the context of ongoing discrimination and stigmatisation as a sexual minority. Pollard et al. (25) extend the significance of the role of discrimination and marginalisation by viewing chemsex through the prism of syndemic and minority stress theories. MSM experience unique marginalisation as Chemsex is situated within three contexts of adversity: (1) prevailing homophobic culture, (2) pressurised and hedonistic gay-subcultures, and (3) interpersonal invalidation. Jaspal (26) also places emphasis on the role of stress which can arise as a member of a sexual minority, but emphasises how these stressors exert themselves through a person’s sense of identity. Drawing on Identity Process Theory, Jaspal (26) argues that sexual-minority stressors harm a person’s sense of self-esteem and efficacy, with chemsex acting as a means by which MSM manage the distress which can result. Ahmed et al. (7) also highlight the social context, but argue that it influences chemsex through social norms - attitudes and beliefs that groups hold, or individuals perceive the group to hold, about appropriate or inappropriate behaviour. Ahmed et al. (7) draw on Social Norm Theory to argue that chemsex emerged in the context of MSM viewing it as normalised among other MSM, how sex was perceived to be different when under the influence of drugs, and in the context of pre-existing social norms around the ubiquity of drug use among MSM.

The second collection of papers applying an explicit theory were predominantly critical of most academic approaches to studying chemsex, which were seen to pathologise sexualised drug use through a focus on health risks. Souleymanov et al. (27, 28) drew on Foucaludian social theory and assemblage theory to highlight how social discourses shape the meaning attached to chemsex, with discourses primarily highlighting pathology, compulsion, and health risks (especially HIV). For Pienaar et al. (22) chemsex is an inherently transformative experience and uses Foucault’s idea of the technologies of the self to argue that drugs are a ‘technology’ that individuals use to transcend themselves and facilitate new subjective experiences. This has particular relevance for gender and sexual minorities where drugs enable the expansion of experiences, possibilities, and pleasure in relation to sexual and gender identity transcending restrictive binary understandings. Similarly, Race et al. (29) highlight the way in which drugs enable a transformation of experience through the transcending of sexual-scripts that structure sexual encounters. Santoro et al. (15) draw on Latour’s theory of ‘black boxing’ to argue that the literature on chemsex has taken the concept for granted, without sufficient scrutiny or discussion around its conceptual parameters. The focus on public health risks potentially downplays the role of important social and cultural dynamics which underpin chemsex. Hakim (11) uses conjectural theory to conceptualise chemsex as an attempt by marginalised communities to experience a sense of collectively which has been undermined through various social, cultural, and historical forces, predominantly a neo-liberal economic paradigm which has eroded queer spaces and emphasised individualism.

Papers that did not use an explicit theoretical framework often framed or positioned chemsex in two distinct ways. The first drew primarily on literature highlighting the health risks or concerns around chemsex, particularly increased risk of STIs, or risks associated with drug use, including addiction and poor mental health. The second approach, however, was often critical of approaches that framed chemsex primarily as a health risk. Instead, these papers explored the social, psychological, and cultural meanings of sex and drugs, as well as how risk was navigated by those who engaged in chemsex.

### Understanding the chemsex experiences of MSM

The search highlighted a range of different themes across the papers, which were broadly divided into four main areas.

#### Theme 1: Characterising chemsex: frequency, settings, drugs, and sexual partners

The first major theme related to characterising chemsex, this included the places and settings that people engage in chemsex, the types of drugs used, the frequency and length of chemsex sessions, the diversity of chemsex experiences. Participants reported using chemsex in the context of group sex, sex with multiple partners, and with individual partners. The length of chemsex sessions varied from hours through to multiple days, and the frequency of engaging in chemsex varied from once or twice yearly (often around large LGBT events), to 1–2 per month or per week, and more frequently.

People who engaged in chemsex reported using a range of drugs. Although crystal methamphetamine, mephedrone, and GHB were the most commonly cited, people also reported using Ecstasy/MDMA, cocaine, cannabis, ketamine, and heroin. Polysubstance use was reported. People also reported using other substances in the context of chemsex, including alcohol and nicotine, amyl nitrate (‘poppers’) and Sildenafil (‘Viagra’). People reported consuming drugs in a variety of ways including injecting, snorting, smoking, and as part of sex acts.

People reported engaging in sexualised drug use in private settings, particularly people’s homes, as well as sex on premised sites, and public spaces, however, it is important to note that some people described chemsex taking place across multiple settings and reported a progression of events. Typically, people described taking drugs for recreational and social reasons in a social setting, such as a club, but as the time progressed and commercial spaces closed, participants would either seek out chemsex, or there would be a progression of going to a private home which developed into chemsex.

#### Theme 2: the context around chemsex

Many participants discussed their experience of chemsex with reference to the wider social and cultural context in which they lived. Reference was made to wider queer culture and the expectations and norms that are perceived by some to be present; the perceived prevalence of drug use generally, and wider social and geographic determinants of gay life; as well as the role of discrimination and minority stress that MSM can experience.

##### Queer culture

This theme captured the wider norms, experiences, and expectations that participants discussed in relation to wider queer culture in which chemsex took place.

###### Apps and hook up culture

When discussing their experience of chemsex, and arranging chemsex, there was an implied normalisation of a wider ‘hook up culture’ which involved meeting people for sex, and the role that digital technology, particularly apps, played in facilitating hooking up and chemsex:

*‘This is the gay life on social media—you are only ever two sentences away from sex or to getting sex.’ (* *30* *)*

*Pressures of the ‘gay scene’.* Often when discussing their experience of online hook ups, there was a recognition by some that it came with pressures and anxieties that could act as stressors in their lives. This often related to fears around participants perceived attractiveness and sexual desirability:

*‘There’s a lot of anxiety that comes with […] dating on Grindr, at least for myself. Dating apps […] can shatter your self-esteem because we all look better in photos than we do in real life.’ (* *31* *)*

Not only was this discussed in relation to online hook ups or dating, but as a more pervasive pressure and fear of negative judgement that existed within queer. There was a perception by some around how current ‘homonormative’ codes of masculinity acted as a distal pressure that was crystalised when participants entered queer space, either physical or online.

###### Minority stress

Participants also discussed external sources of stress that could be characterised as minority stress. Some participants spoke of how they continued to experience stigma and discrimination because of their sexuality. Whilst the gay scene could come with its challenges, there was a recognition that it offered a space where people could express themselves and their sexuality in a way that was not possible in a heteronormative world.

###### Changing nature of gay spaces and ways of socialising

Many participants spoke about the changing nature of queer spaces and the ways in which MSM socialise. Participants reflected on a shift from clubs and public spaces toward meeting in private settings, such as people’s homes, often facilitated through use of apps, leading to a sense of the diminishing relevance of clubs, bars, and pubs as spaces for MSM to meet:

*‘… people don’t need to go out to meet people. […] because we’re all living in Lambeth so we all fucking know each other. We can all get each other on [app]. There’s a dealer there, there, there and there. We don’t need the clubs.’ (* *7* *)*

###### Geography

Implicit within some people’s accounts of their chemsex use was the role of geography, and in particular the importance of the city and gay spaces within it, as either the context in which chemsex happens or as a distal driver behind people’s use. These gay spaces seemed important in attracting a large collection of MSM people, as well as bars, pubs, clubs, and sex-on-premises venues, which facilitated access to other MSM people, improved access to drugs, as well as fostered the cultural context in which drugs and sex was commonplace and accepted.

*‘… then you come to Vauxhall and you’re just hanging out, there’s just so much temptation, and you go on Grindr and there’s a really good-looking guy inviting you over and you just kind of fall into the circle of people and behaviour. It becomes something you enjoy and then it becomes hard to get out.’ (* *25* *)*

###### General drug use

Whilst drug use was predominantly discussed in relation to sex, some participants also spoke about their use of drugs more generally, including the changing and varied nature of their drug use, and how their use of drugs was not solely in relation to sex.

*‘I suppose I've grown up on a culture of ecstasy and, obviously, speed and acid and that, so that was back in the day. That's the reason why I would take it, you know, because of the lights and the music and people and then it, sort of, just gradually over the years been into a sort of sexual thing.’ (* *8* *)*

Participants spoke about the use of different drugs in different settings for different reasons, such as using cocaine or MDMA when going to nightclubs so that people could dance or stay awake for longer, or at friends’ homes to enhance social interactions. For some, the setting influenced the experience of the drug itself, such that the use of methamphetamine in a club rarely led to sexual activity as well as shaping what drugs were seen as acceptable and how they were taken, for example ecstasy/MDMA or cocaine were seen as more socially acceptable in a club compared to injecting crystal methamphetamine. There was also a sense that preferences for drugs naturally changed over time, with some discussing how there was a shift from alcohol as the dominant substance to ecstasy/MDMA and cocaine to GBH/GHB through to mephedrone and methamphetamine. The introduction of new drugs intersected with or contributed to the changing motivations for drug use:

*‘Before, we used to go out, and we’d take drugs to keep going at after-parties. It was for partying. Sex wasn’t the main reason.’ (32).*

#### Theme 3: the chemsex experience

##### Enhanced sexual experiences

There was an overwhelming sense that the sex was better when using drugs –it lasted longer, felt better, and there was a sense of the overall experience as more intense, physically, psychologically, and sexually. For some, the pleasure was almost indescribable and meant that chemsex was somehow inherently different or unique compared to sober sex

*‘[on mephedrone] It was the best sex I ever had. Really the best orgasm I’d had. I used to say it was like the heavens opened and it was like the light came down when I had an orgasm. Because it was that intense on drugs, it really was, I’ve never experienced that sober’. (* *13* *)*

##### Physiological aspects of chemsex

In describing the increase in pleasure and satisfaction with sex, participants mainly spoke of the ability to perform better physically and having significantly increased libido

*‘you feel you want to have sex all the time, you like that. With no effort, you have a great libido, you want to fuck everyone.’ (* *32* *)*

Physical performance related to how drugs improved stamina and increased energy levels, helped delay orgasms as well as the ability to have multiple orgasms. The improved physical performance and increase in libido were not simply convenient by-products of drug taking, but for many was greatly valued and an important reason behind their drug use:

*‘[I use crystal methamphetamine] for sex… Definitely you can go longer—definitely I have had 11–15 hours sessions—and it makes you want more and you are turned on totally. Or at least I am…I use it definitely for sex!’ (* *20* *)*

For a minority, the drugs enabled them to overcome health-related challenges that impaired their sex lives, including loss of libido, pain and fatigue, the effects of aging, and to counteract the side effects of HIV medication.

*‘I don’t have a sex drive any longer. It’s one of the reasons why I started slamming chems because when I slam, I get horny.’ (* *13* *)*

##### Psychological aspects of chemsex

Many participants described how the use of drugs allowed them to ‘let go’ and push their limits sexually

*‘I had a crazy good time with slam [injecting drugs, typically crystal methamphetamine]. Even in regard to my sexual practices, there’s no more barriers, you always want to push your limits further, but there are no limits.’ (* *9* *)*

Participants described how they were more able to explore their sexuality in an almost completely unimpeded way. Participants reported that they felt sexually less inhibited and able to engage in more diversified sexual practices, which for many would not have felt possible sober People also reported an improved sense of sexual and psychological self-confidence.

The psychological advantages that drugs conferred also meant that the relational aspects of sex and romantic experiences were intensified. Chemsex allowed for the intensification of romantic experiences and shared intimacy between people:

*‘Having him in my arms, suddenly I was comforted and I felt so light. It was a feeling of complete well-being. He was cute, and thoughtful. I was too. We pleasured each other and we only wanted the best for one another. It was a special moment.’ (* *33* *)*

##### Psychological motivators for chemsex

Many participants revealed that they carried some sexual, inter, and intrapersonal insecurity. In particular, how low self-esteem and insecurity about their body and perceived desirability had a significant negative effect on their ability to initiate and enjoy sex:

*‘I think when I was using drugs I did not have body issues. I did not think, I am feeling a bit too fat or feeling that I do not really feel that attractive so it reduces inhibitions physically and psychologically in terms of having sex and with people you would not feel comfortable, like, having sex with normally.’ (* *13* *)*

These anxieties could become crystalised or brought to the forefront when navigating hookups (sex) with people they had not met before, and drugs allowed for the management of these concerns:

*‘there’s a lot of anxiety that comes with actually meeting the person and, you know, expectations. At least for me, I worried a lot with, like, “Oh, what if they don’t think I look like I’m the same person?” Or, like—and I know my friends also have gotten really bad comments. Like, “Oh, you’re bigger in person,” or things like that […] I think I would need help from … some substances […] to get over those anxieties […] about the sex […] because when you talk about trust and you … I think alcohol and I think weed and stuff like that, it kind of just skirts those problems away. […] It’s not an issue anymore.’ (* *31* *)*

Some participants recognised that chemsex was a type of escapism, allowing them to escape from the pressures and banality of life At the more extreme end, participants recognised that chemsex was used as a means of managing or overcoming psychological distress The nature and cause of the psychological distress varied, with participants reflecting on their sense of loneliness, depression, the emotional consequences of ending a relationships, work and family stresses, and managing the long-term consequences of trauma and marginalisation as a sexual minority.

##### Perceptions, experiences, and expectations

##### Perceived prevalence

Many participants reflected on how widespread they believed chemsex was within MSM communities. Whilst the perceived prevalence was not universally shared, some people perceived chemsex as normalised, with some even perceiving it as an inherent part of what MSM men do.

*‘It is a kind of gay men aggregation rite involving sex and drugs.’ (* *34* *)*

###### Initiation into chemsex

Participants spoke about the variety of ways that they started using drugs when having sex A common narrative related to the offer of drugs through sexual partners, but also friendships, and being in situations, such as saunas or parties, where they were offered drugs with the intention of having sex. Many participants also described a sense of curiosity around chemsex, with the decision to try it either a conscious decision or more opportunistic in nature, often as a result of being offered drugs by a sexual partner. Some participants spoke of more significant personal stressors or events, such as the end of relationships, or moving to a new city. People’s relationships with drugs before chemsex varied, some had not used drugs at all, whilst others had much more developed drug histories and saw new chemsex-related drugs as the evolution of their drug use.

###### Organising chemsex

Many people discussed how chemsex was organised. In particular, participants discussed the role of digital technology, including using geo-spacial networking apps as a means of accessing drugs and sexual partners. Alternatively, participants would go to saunas, and some people discussed how chemsex was organised through existing social networks

*‘Right now I probably have a network of around 30 people in Madrid who I can meet to practise chemsex with, and they're guys I've known for three years, and in turn we have other people we know in common.’ (* *15* *)*

###### Relationship between sex and drugs

Within some participant narratives, a distinction was made between those whose use of drugs was primarily sexual and those whose sexual effects were secondary or only one reason for why they used drugs.

*‘Like, I had sex on gear, but it was never like “let's have sex and gear”.’ (* *8* *)*

Although some participants clearly saw drugs and sex as inextricably linked, this was not universally shared, and participants described a more fluctuating and variable relationship between their use of drugs and its relationship with sex. Some spoke of a gradual transition in their desire to use drugs for sex, this was expressed both longer-term as well as over the course of a single evening. For some they derived pleasure in taking drugs and this was a more primary motivator behind their drug use, for others, it is a means of enhancing social situations, improving concentration, and maintaining energy levels.

#### Social aspects of chemsex experiences

Within the narratives of participants was the importance of the social aspects to chemsex, where MSM men could meet, develop or continue friendships, dance, and relax together. Central to this was the idea that chemsex settings often offered people a space of acceptance, safety, belonging, and community.

*‘The feeling of sharing, of coming together with others, belonging to a group that recognises itself as a group.’ (* *32* *)*

#### Theme 4: harms, saying safe, and stopping chemsex

##### Harms and risks

Within the narratives of participants, there was a recognition that chemsex came with potential risks as well as acknowledging that it had caused harm to themselves and/or others. *Differing recognition of needs and outcomes.* For many participants, there was recognition that the potential risks or harms associated with chemsex varied and severity. Harm and risk were complicated ideas that, for participants, were predicated by a range of factors, such as the types, frequency, and quantities of drugs used and the method of drug delivery, how actively ‘in control’ they were over their use, and the types of sex that they engaged in.

###### Coercion, consent, and sexual assault

A major theme within the narratives of participants related to witnessing examples of coercion, ambiguities around establishing consent, and sexual assault Reports of coercion often relate to subtle use of power to encourage people to acquiesce to sexual practises and to take drugs. For some there was an ‘economy of drug use’ where people would engage in certain sexual acts with others in exchange for drugs. The extent to which this was an equal decision by two people was not clear, but came with an implied sense of subtle coercion. Establishing and withdrawing consent during chemsex could be complicated by the effects of drug use on cognition and decision making.

###### Loss of control

Participants spoke of the potential for or experience of loss of control over chemsex. This loss of control was often characterised in relation to escalating and excessive drug use and chemsex to the point that it either dominated their lives or negatively impacted multiple aspects of their lives. For some, chemsex could become ‘all-consuming’ to the point that it was difficult to stop or remain in control. Some participants reported how drugs could lead to a loss of consciousness, which would vary in how long it would last.

###### Physical, psychological, social, and occupational harms

In discussing the negative psychological consequences of their drug use, participants reported a range of problems including difficulties associated with the aftermath of drug use (‘comedowns’) including low mood, lethargy, feeling run down, vulnerable, and questioning their decision, along with feeling on edge, highly anxious, depressed, suicidal, as well as paranoia, auditory and visual hallucinations, and psychosis

*‘The after … the sense of guilt and emptiness … I had thoughts of death…’ (28yo, predominantly gay) (* *35* *)*

Some participants also felt that their use of chems had a negative effect on their relationships, both friendships and romantic, this could include the loss of relationships, the end of romantic relationships, and difficulties establishing a romantic relationship. Problematic chemsex could also have a negative effect on people’s work, such that they struggled to concentrate and missed work or education. Participants also discussed the negative effect that chemsex had on their physical health. Some of this was related to the cumulative effects of heavy drug use, but more typically it was related to physical damage due to of sex and drug use. Participants reported rectal trauma or penile abrasions and drug-related damage included collapsed veins and muscle damage.

###### Sexual health and satisfaction

The negative consequences of chemsex in relation to participants sexual lives varied considerably from bad, painful or unsatisfactory sex to engaging in risky sexual practises, contracting STIs, and sexual dysfunction. Some participants spoke of how bad, painful, and unsatisfactory sex could be while on drugs. Participants spoke of a type of ‘sexual selfishness’ that meant the qualities of sex that they valued, such as heightened relational and emotional connection, were not obtained. Instead, some participants reported experiences where the desire for gratification was at the expense of heightened emotional intimacy that was a key motivator for engaging in chemsex. Many participants discussed how drugs could result in sexual dysfunction, particularly difficulties in getting or maintaining an erection.

Although some reported regret or concerns about engaging in riskier sexual behaviours when having chemsex, this was not universally shared. Some participants reported how their perception of risk and what they were willing to do changed over time, such that they had made the decision to not use condoms, which was unrelated to their drug use. In addition to this, others were of the belief that contracting STIs was not a significant concern. This took on added significance in the context of the use of pre- (PREP) and post-exposure prophylaxis (PEP) for preventing HIV.

##### Staying safe

###### Individual harm reduction strategies in relation to drugs

*P*articipants reported a range of strategies that they engaged in to reduce the risk to themselves. By far the most common strategies related to limiting the frequency and timing of chemsex and setting limits on their drug use, including limiting the type and/or quantity of drugs they used. Participants also reported a range of safe drug practises, including using clean needles, not sharing needs, or use of individual straws to snort drugs.

###### Shared responsibility when taking drugs

Participants also spoke about a shared sense of responsibility and mutual help when it came to drug use. This often involved looking out and caring for those who had perhaps taken too many drugs, having people more experienced with certain drugs supporting those who were more novice users, as well as having shared responsibility and awareness of what drugs people had taken, when, and in what quantity through a written or electronic document that recorded the names, times, and quantity of drugs taken by individuals.

###### Sexual health

Participants reported using condoms when having sex, having open discussions about their sexual health status which drugs sometimes facilitated, as well as using PREP and PEP to limit the risk of contracting HIV. Whilst participants were aware that the drugs could influence how stringently these practises were adhered to, participants reported being able to stick to their boundaries around what types of sex they wanted and doing it in a way that they felt limited their risk

*‘He said, “Listen, you can’t fuck me with that dick because it’s got a condom on it.” […] And I said, “I’m always going to wear this condom, no matter what.”’ (* *36* *)*

##### Stopping chemsex

When it came to stopping chemsex, participants reflected on how challenging this could be. There was an awareness of how much they liked having chemsex alongside the harms that it could cause, meaning that for some, they were unsure if they could, or wanted, to stop. While there was little research into relapse, a few participants spoke of how they would have a period of deliberate abstinence, but this would not be sustained, and they would relapse. Whilst there was no substantial literature around life after stopping chemsex, there was a narrative of needing to mourn what was lost from stopping. In particular, participants spoke of mourning their sexuality and the difficulty of having sober sex, which could not match the intensity of chemsex sessions. Stopping chemsex also required a significant change to, potential loss of, and need to rebuild romantic relationships and friendships that were not connected with chemsex.

##### Seeking professional support

When discussing chemsex, some participants discussed their experience of seeking professional support and/or the type of support they felt they needed. In discussing their experience of seeking support around chemsex, some participants discussed issues with struggling to access services. For some, this was related to simply not knowing where to go for support. While some participants mentioned mental health and drug addiction services, there were concerns about whether these services were appropriate for their needs. The most consistent concern reported by participants was that traditional services lacked cultural awareness around both MSM health needs and sexualised drug use, which could result in some feeling judged either for their drug use or sexuality, or needing to explain chemsex to professionals who lacked an understanding of the interaction between drugs, sex, and MSM health needs.

When discussing the type of support required, participants recognised the need for more support that was culturally appropriate and integrated spanning sexual, physical, and mental health as well as drug addiction, offered clear and accessible information, recognised the varying health needs of those who engage in chemsex, and was delivered in a non-judgemental manner. Whilst professional support was valued, there was a sense from some participants that support also came outside health services, including community-based organisations.

## Discussion

This is the first systematic review of qualitative studies of chemsex experiences of MSM and the theoretical frameworks that have been used to conceptualise chemsex. Past reviews (4–6) have primarily focused on quantitative research, with an emphasis on understanding key characteristics and health consequences of chemsex. Although Maxwell et al. (5) review explored the chemsex participants’ expectations, this focused on proximal expectations, particularly in relation to the effect of drugs on sex. A mixed-methods systematic review by Lafortune et al. (66) examined qualitative research, but was limited to psychological and interpersonal factors around chemsex and only included a limited number of studies.

Some of the results confirm findings from previous systematic reviews. In understanding the emergence of chemsex as a phenomenon, research focused on characterising chemsex in relation to the types of drugs use, how they are consumed, where chemsex takes place, the number of sexual partners, and the role of technology in facilitating chemsex. However, the key findings from our review also highlight the importance of situating chemsex within a wider social, cultural, and geographic context. This includes distal factors such as the changing nature of gay or queer spaces and the evolution of the means by which MSM communicate, socialise and congregate. The emergence of digital technologies has intersected with other cultural factors within queer culture, including casual sex (‘hook up culture’), general drug use, and for some, repudiation of hetero-and homo-normative expectations (11, 37). Although chemsex has been seen as a distinct phenomenon, it is important to situated its emergence within pre-existing social and cultural dynamics.

The results of this review add evidence to the claim that a central proximal motivator for chemsex is the effects that drugs have on perceptions of sex, both physically and psychologically (5). However, our results also suggest that there are complex and multiple psychosocial factors that may directly and indirectly influence why a person may have chemsex, which go beyond the immediate effects of drugs on perceptions of sex. Psychologically, some people who have chemsex reported how it helped overcome low self-esteem, insecurity about their body and perceived desirability, anxieties about sex, and psychological distress. This was also in the context of need for acceptance and belonging from both within and outside of the queer community, highlighting the complex struggles for some of navigating life as someone from a sexual minority and the potential role of structural social issues including marginalisation and homophobia. Importantly, there is an absence of research which explores these issues from an intersectional lens, including research among gender and ethnically diverse populations given the unique challenges these populations can face (38). At present, there is limited research beyond qualitative studies (66) that explore a broad range of psychological factors that may confer greater risk, either as a risk factor for problematic chemsex and/or negative health outcomes, or act as protective against such consequences. Research has predominantly focused on the relationship between mental health problems, particularly anxiety and depression, and chemsex (39), but there is a need to broaden the focus to look at a range of other psychological factors, including issues with body image, self-esteem, community and belonging, sexual insecurities, and strategies for managing psychological distress. There is emerging evidence that for some people, chemsex is a strategy for managing emotional dysregulation that arises in the context of adverse childhood experiences (40, 41). Our findings, combined with this fledgling literature, suggest that there are complex and multifaceted psychological drivers behind chemsex, which require further exploration. Furthermore, given that not all chemsex is problematic, identifying the individual psychological differences between those with problematic versus non-problematic use would help to identify areas of resilience that may be protective against negative consequences.

The findings also help to broaden the perspective on supporting the health needs of those who report chemsex. Consistent with previous reviews, we found that there can be negative health consequences of problematic chemsex in relation to mental health, substance misuse and loss of control, increased risk of STIs, dependence on drugs, and negative physical effects from cumulative heavy drug use (4–6). However, our findings highlight that people adopt a range of individual and collective strategies to reduce harm and risk in relation to drugs. Furthermore, risk in relation to drugs and sex had different meanings for different participants, and perceptions could change over time. This has added significance in relation to the prevalence of pre-pre-exposure prophylaxis (PREP) and post-exposure prophylaxis (PEP) for HIV. Understanding the individual experiences and decision making of people in relation to ‘health risks’ has important implications for how people who have chemsex are supported. There is a recognition within the wider chemsex literature to move beyond a ‘risk and health outcomes paradigm’ (8, 41, 65) and adopt an approach that recognises the resilience of those who engage in chemsex (69), including recognising and promoting the strategies used to reduce potential harm. Finally, this review brings together qualitative research on how people stop using chemsex and seek professional support for their use, which remains an under-researched aspect of chemsex. Supporting people to stop or move toward safer chemsex needs to consider a broad and holistic understanding of needs (42), including issues of wellbeing, psychosexual factors given the potential challenges of sober-sex for some people, and help to develop social networks that foster a sense of belonging outside of chemsex (43). Health services and communities that may work directly with or encounter people who engage in chemsex need to be supported in their understanding of the needs of this population, with suitable cultural understanding and awareness. This highlights the need for further research into how this was achieved and what interventions could be developed to support people who wish to stop or to practise chemsex more safely (44).

The second aim of this review was to explore the conceptual and theoretical paradigms adopted in exploration of chemsex. Although chemsex can be viewed through different disciplinary and methodological lenses, much of the qualitative literature omitted reference to underlying theoretical perspectives that informed their conceptualisation of chemsex as a phenomenon. Nevertheless, many of these papers framed chemsex within a health and risk paradigm, raising concerns about the potential negative health consequences. While acknowledgment of the potential health implications of problematic chemsex is required, it is important that a theoretical understanding of who, when, and how chemsex may become problematic is incorporated. Furthermore, a more sophisticated theoretical understanding of chemsex, which incorporates a range of social, cultural, and psychological factors, is needed to guide research and inform the response to chemsex.

### Implications

The findings of this review have implications for those working with people who engage in chemsex. There is now substantial evidence that a primary motivator for having chemsex is how it enhances sex, yet the motivation to have chemsex is influenced by other psychological, social, and cultural factors. This necessitates a comprehensive assessment of people’s needs that includes an individualised assessment of the perceived benefits, drivers, and consequences, that can come with chemsex. This review suggests that important psychological factors - namely concerns around body image and self-perception, often in the context of ongoing marginalisation as a sexual minority group - may reflect important areas of assessment, and potentially intervention, drawing on evidence-based interventions at both the individual and public health levels. Participants highlighted the challenges that came with accessing support, principally confusion about where to seek help, and feeling that services were not fully able to meet their needs. This raises important questions about how best to develop services for people seeking help related to chemsex. There is now a recognition of the need for chemsex-specific services to be developed, either as standalone services or embedded within existing services, that offer comprehensive and tailored services that can meet the psycho-sexual, mental health, substance misuse and sexual health needs of people who have chemsex (45, 46); this includes the need for co-production of service design and delivery, often in tandem with existing charity and third sector organisation that have a trusted reputation amongst the community (45). Future research should focus on understanding pathways into chemsex, how and when it becomes problematic, and how best to support people when chemsex is associated with problems typically associated with polysubstance misuse (47). Finally, there is a long history of using a range of theories, in particular those from the behavioural sciences, to understand and support MSM with regard to their health and wellbeing, yet chemsex remains an area of under theorised.

### Strengths and limitations

One strength of this review is the narrative synthesis using qualitative literature only. This is important because understanding the theoretical frameworks that conceptualise chemsex provides a basis for consistency across future research and can be utilised for designing training to provide to professionals. At present, there is a notable absence of a theoretical understanding of chemsex, as highlighted by the small number of studies that explicitly stated their theoretical framework.

One potential limitation is that the review included literature in English only. This reduces the scope of the review and the impact and reach that it may have. The review only included MSM, thus limiting the transferability to other people who engage in chemsex.

## Conclusion

This is the first systematic review of the qualitative literature on the chemsex experiences of MSM and the first to explore theoretical frameworks that have been used to conceptualise chemsex. The results add further understanding of some of the key characteristics of chemsex, while highlighting important psycho-social factors that inform why people engage in chemsex, as well as addressing specific healthcare care needs that may need to be met. Further research is needed to better understand the psychosocial factors that may shape why people have chemsex, their experiences, and outcomes.

## Data Availability

The original contributions presented in the study are included in the article/supplementary material, further inquiries can be directed to the corresponding author.

## Appendix

**Table:**
